# Practical Issues in the Treatment of Preterm Infants

**DOI:** 10.3390/children10050849

**Published:** 2023-05-08

**Authors:** Shmuel Arnon

**Affiliations:** 1 Department of Neonatology, Meir Medical Center, Kfar-Saba 4428164, Israel; shmuelar@clalit.org.il; 2 Sackler School of Medicine, Tel-Aviv University, Tel-Aviv 69978, Israel

Each year, an estimated 15 million babies are born too early; more than 1 in 10 babies. Approximately 1 million children die each year due to complications of preterm birth [1]. Many survivors face a lifetime of disability, including learning disabilities, and visual, hearing and mobility problems. Current research involving preterm infants is focused primarily on clinical improvement and on supporting these infants, their caregivers and medical personnel by introducing modalities of developmental care. 

In this Special Issue of *Children* concerning “Practical issues in the treatment of preterm infants”, clinical and developmental care are very well represented. In a comprehensive paper, Dall’Oglio et al., on behalf of the Family-Centered Care (FCC) of Italian NICUs Study Group, showed that by using the FCC Revised Questionnaire, profession, education level and work experience seemed to positively influence the perception of what is required for FCC practice within NICUs [2]. In a study examining an assessment tool of decision-making group dynamics between groupthink and polythink in matters involving preterm infants, Riskin Y et al. showed that the tool can define a method of group thinking and may help achieve better decisions by adopting other methods of group decision-making [3]. Riskin A et al. presented a novel method of communicating with parents during a period of restricted visitation, such as during the COVID-19 pandemic. They found that parents were comforted when they received a daily video recording of their infant in an incubator or cot via the WhatsApp application [4]. Pain, one of the main stressors of preterm infants during their stay in the NICU, was the subject of a paper from Brazil. The authors showed that during suctioning, both gentle touch and sucrose reduced pain, with no difference between the two techniques [5]. In a systematic review examining the effects of a rehabilitation intervention on the emotional regulation of children born preterm, Dell’Aversana et al. showed that parent training and mindfulness interventions can be helpful rehabilitation techniques for children born prematurely. However, they also recommended additional research on establishing interventions [6].

Regarding the clinical aspects of treating preterm infants, a group from Taiwan demonstrated that small-volume blood sampling resulted in lower PRBC transfusion volumes, less severe anemia, and greater bone marrow function at 30 days of age [7]. By reporting their local experience, Riskin et al. showed that preferring central IV access for preterm infants born at ≤32 weeks or with a head circumference ≤29 cm resulted in decreased need to insert IV lines. The authors recommend that other NICUs should study their own data and draw their practice guidelines for preferred IV access (central vs. peripheral) upon admission to the NICU [8]. Chetta et al. demonstrated that delayed receipt of antibiotics was more common in infants with surgical rather than medical necrotizing enterocolitis. They recommended that larger studies are needed to investigate whether optimizing antibiotic expediency may improve intestinal outcomes [9]. In a study analyzing 127 very-low-birth-weight premature infants, the process of weaning from an incubator was shown not to adversely affect gains in body weight or calorie intake [10]. 

Two studies in this Special Issue investigated the relationship between prenatal findings and neonatal outcomes. By describing fetuses with cleft lip and/or cleft palate and their neonatal outcomes, Farladansky-Gershnabel et al. suggested that additional work-up of these fetuses should be provided, as well as improvements in parental counseling and their understanding and compliance regarding postnatal therapeutic planning [11]. In another study, the same group of investigators showed that pyelectasis of 6–9.9 mm in fetuses remains stable or resolves spontaneously during pregnancy. Postnatally, renal reflux and/or renal obstruction might occur in this group; however, most infants do not require surgical intervention [12].

External cephalic version (ECV) is a safe, cost-effective option for breech presentation at term. A study using Doppler demonstrated that ECV is associated with changes in the Doppler indices of the umbilical artery that might reflect interference with placental perfusion. These changes are probably short-term and have no detrimental effects on the outcomes of uncomplicated pregnancies [13]. Sirota et al. showed that continuous vs. bolus feeding decreases regional gut tissue saturation but does not affect oxygen extraction by intestinal tissue. This may indicate that the mode of enteral feeding should be individualized and adjusted to the hemodynamic, respiratory and nutritional status of each infant [14]. In a laboratory-based study, Levkovitz et al. showed that maternal vitamin D levels correlate with cord levels but do not affect bone strength or growth parameters [15]. 

Music therapy is a developmental care modality used in the NICU to reduce infant and parental anxiety and to augment parent–infant bonding. A study about music therapy by Arnon et al. demonstrated decreased noise and increased signal-to-noise ratio, indicating that music therapy is feasible even in a noisy NICU [16]. The last manuscript published in this Special Issue involved maternal SSRI treatment during pregnancy that was terminated prematurely. The results demonstrated very clearly that no differences in the total duration of respiratory support, time to reach full enteral feeds, length of stay or complications of prematurity were associated with this situation [17]. 

In conclusion, the scientific methodology regarding the treatment of vulnerable preterm infants is rapidly advancing. Further Special Issues dealing with preterm infants are warranted.

## Data Availability

Not applicable.
